# Public Health System Economic Efficiency and COVID-19 Resilience: Frontier DEA Analysis

**DOI:** 10.3390/ijerph192214727

**Published:** 2022-11-09

**Authors:** Aleksandra Kuzior, Mariia Kashcha, Olha Kuzmenko, Serhiy Lyeonov, Paulina Brożek

**Affiliations:** 1 Department of Applied Social Sciences, Faculty of Organization and Management, Silesian University of Technology, 41-800 Zabrze, Poland; 2 Economic Cybernetics Department, Sumy State University, 40000 Sumy, Ukraine; 3 The London Academy of Science and Business, 120 Baker Street, London W1U 6TU, UK; 4 JSofteris, 41-219 Sosnowiec, Poland

**Keywords:** frontier DEA analysis, health system, efficiency, CCR model, COVID-19 pandemic, human, infectious diseases, public health

## Abstract

The article summarizes the arguments and counterarguments in the scholarly discussion about the problem of choosing a model of healthcare organization. The study’s primary goal was to identify the economic efficiency of the public health system and resistance to COVID-19. The relevance of addressing this research issue is that the epidemiological challenges posed by the pandemic worldwide have manifested themselves differently in various countries. Therefore, it is advisable to consider the effectiveness of public healthcare models and how they have worked out in the fight against COVID-19. Research in the work was carried out in the following logical sequence: conducting scientometric analysis of research, creation of a statistical research base for 22 countries of the world; construction of integral indices of the economic efficiency of the health care system; calculation of public health system resilience to the COVID-19 pandemic; application of frontier DEA analysis to determine system efficiency; comparison and analysis of the results of research on the economic efficiency of public health systems obtained by different methods. The article presents the results of a comparison of the economic efficiency of the public health system, which showed that the system built according to the Beveridge principle is the most resistant to the pandemic and, at the same time, has the highest indices of economic efficiency.

## 1. Introduction

The COVID-19 pandemic has shaken the world with its speed of propagation and the complexity of post-COVID implications. There is not a single country left around the world that has not been affected by the pandemic financially and human losses directly. Gains in global production have grown smaller and smaller every year; the world’s most powerful economies, such as the USA, China, and the European Union, are no exception [1]. According to the total number of deaths caused by the COVID-19 pandemic, from the beginning of the pandemic to August 2022, the world leaders were Peru, Bulgaria, Hungary, Georgia and Montenegro. If financial losses are considered, the USA, India and Brazil suffered the most significant losses, according to the UN World Economic Situation Report 2022 [1]. Conversely, over the same period, New Zealand, Thailand, China and Australia suffered smaller losses. 

Thus, the question arises: Why have different countries suffered different degrees of impact from the world pandemic in terms of the number of deaths, the number of individuals infected, the disease propagation rate and the magnitude of economic loss? Regarding economic loss and the disease propagation rate, one of the reasons is the different responses of governments and the stringency of quarantine measures imposed. In particular, if we compare the duration of the lockdown, it was different; for example, in Estonia, it lasted only 31 days (from 11 March 2020 to 11 April 2020) and in Greece, it lasted for a combined duration of 177 days (from 23 March 2020 to 4 May 2020 and from 7 November 2020 to 22 March 2021) [2]. Moreover, the lockdowns were implemented at different levels—from individual cities to entire countries—and also had a differential impact on small- and medium-sized businesses, which suffered significant losses. If we look for reasons for the different numbers of infected per 1000 population, one answer might be different communication cultures. In particular, in Japan, the population was accustomed to wearing masks, Japanese people bow instead of shaking hands when meeting each other, and were more responsible about maintaining social distancing, even though it was not forbidden to go out and patronize public food and entertainment establishments [3]. Unlike Japanese residents, Italians are used to greeting their acquaintances not just with a handshake but with a kiss, and also painfully endured the closures of cafes and theaters [4].

Thus, given the different policies of the governments of the countries, different attitudes of the population regarding restrictions might explain the varied impacts of the pandemic. However, if we compare the proportion of seriously ill and fatal cases to the total number of infected, the question remains: Why do different countries cope differently with the burden on the healthcare system? The hypothesis of this study is that differences in accessibility of the population to healthcare, the quality of medical services provided, and the amount of spending on the healthcare system have influenced the ability to treat COVID-19 patients and provide medical services to all those in need without exception. It is this combination of the factors mentioned above that gives a comprehensive picture of the model of the healthcare system organization.

The goal of our research was to identify an effective model of healthcare system organization that will be more resistant to current and future epidemiological challenges, which, in addition to saving precious human lives, will protect the world economy from significant losses.

## 2. Literature Review

The COVID-19 pandemic has changed the vectors of scientific interest for researchers worldwide. In particular, studies that look for strengths and weaknesses, such as those related to the construction of public healthcare systems that made it possible to counteract the consequences of the pandemic effectively, have become popular. For example, studied the behavioral intentions of the population to get vaccinated and predicted the consequences of the speed of the spread of the virus [5]; looked in detail at tools that help increase the effectiveness of public health in EU countries [6]; in researchers developed road maps for possible future epidemiological threats to ensure public health at a sufficient level [7]; while investigating the problem of child mortality, identified a particular set of stabilizers in the organization of health care [8]; considered the role of mass media in covering the actual situation with the pandemic [9]; saw a relationship between the duration and severity of quarantine on the one hand, and the speed of disease spread on the other [10]; examined the degree of protection of doctors against diseases while working with patients [11]; came to the conclusion that due to the additive economy, namely the increase in production efficiency, it is possible to achieve a reduction in social risks, including in the medical field [12].

Special attention should be paid to studies in which the problems of medical institutions occupy a special place and significantly reduce the efficiency of the entire medical system nationwide. In particular, studied the labor market of medical workers and proved that it was significantly affected by the impact of the pandemic [13]; based on the experience of the USA, considered the differences in actual medical protocols [14]; studied labor resource management mechanisms in hospitals and other medical institutions [15]; also based their research on labor resources and their transformation during the pandemic [16]; and examined the quality of public service in terms of the provision of medical services, which catalyzed improving the efficiency of the medical system during the pandemic [17].

Additionally relevant are studies that highlight public awareness as a necessary condition for social responsibility for one’s health and the health of others. For example, explored the relationship between the pandemic and digitization [18,19], which is capable of reforming the management system in any field—including medical [20,21,22]; saw problems in the imperfection of marketing activity in terms of medical products, which complicates the timely treatment of the population, due to widespread self-medication, including during a pandemic [23]; insisted on the need to popularize a healthy lifestyle around the world as the main tool for improving the general level of public health [24]. noted that the social responsibility of tourists is a central research problem because migration processes and the tourism industry influence the spread of disease quite strongly [25,26,27].

Due to the accumulation of specific problems in the socio-economic development of individual countries and the world at large, the consequences of the pandemic manifested themselves in different ways, drawing scientists’ attention worldwide. In particular, saw that due to the pandemic, there was an accumulation of crisis phenomena in economic and social sectors that negatively affected the economic security of the whole world [28,29]; cited the development of the digital economy during a pandemic [30]; cited changes in the labor market [31]; proved the negative impact of the pandemic on the expenses of Ukrainian households due to the closure of borders and a decrease in cash flows into the country [32]; in the researchers described in detail the problems that existed in the demographic sphere of many countries worldwide, which became the cornerstone of communication in the challenges of the pandemic [33]; in scientists discovered which sectors of the national economy and the socio-economic sphere needed the most attention from the government in the implementation of innovative policies due to the consequences of the pandemic [34]; and proved that the pandemic catalyzed systemic supply chain problems for sizeable multinational manufacturing companies [35]. So, it is undeniable that the COVID-19 pandemic has changed many different spheres of life in global society; researchers all over the world have changed the vectors of their research to connect the causes and consequences of the pandemic. That is why research is also relevant, as it can help to establish cause-and-effect relationships between gaps in socio-economic development, the model of organization of the health care system, etc., and the course of the pandemic.

Attention should be paid to studies in which the authors searched for cause-and-effect relationships within the chain—between the pandemic, economic development, and the ecology of the region. In particular, proved that the introduction of the Dorst quarantine stopped the spread of the virus, but had a detrimental effect on the macroeconomic development of countries and their ecological development [36]; saw the prompt response of national governments to epidemiological and environmental challenges as a necessary condition for their respective countries’ sustainable development [37]; proposed an optimal level of environmental taxation that would ensure countries sustainable development while protecting them from epidemiological threats [38,39]; added energy security to economic and environmental, as it also was affected by the impact of the pandemic [40]. 

Another aspect of this study was to determine the economic efficiency of health care organization models, which was also the subject of a large number of other scientific studies. In particular, conducted a thorough study of various methods for assessing economic efficiency, including frontier analysis, Bayesian methods, analysis of asymptotic properties, etc. [41]. The concept of economic efficiency is used in various fields: in the energy sector, comparing different types of lighting [42]; in the water supply sector [43]; and in the purely economic branch by focusing on the concept of economic efficiency to achieve economic sustainability [44]. There are also a large number of methods for the determination of model efficiency. For example, in DEA analysis was applied to assess the economic efficiency of medical laboratories, given the costly laboratory equipment [45]; carried out a study of economic efficiency through a survey among medical personnel within one medical institution, which found that medical personnel aware of the fundamental principles of economic efficiency did not believe this should be the first issue in clinical practice [46]. Consequently, the concept of economic efficiency is widespread among scientists and is applied in different spheres of research; therefore, it makes sense to apply it for research on the efficiency of various health care organization models worldwide, both in the form of an integral indicator and in applying it to frontier DEA analysis.

**Figure 1:**
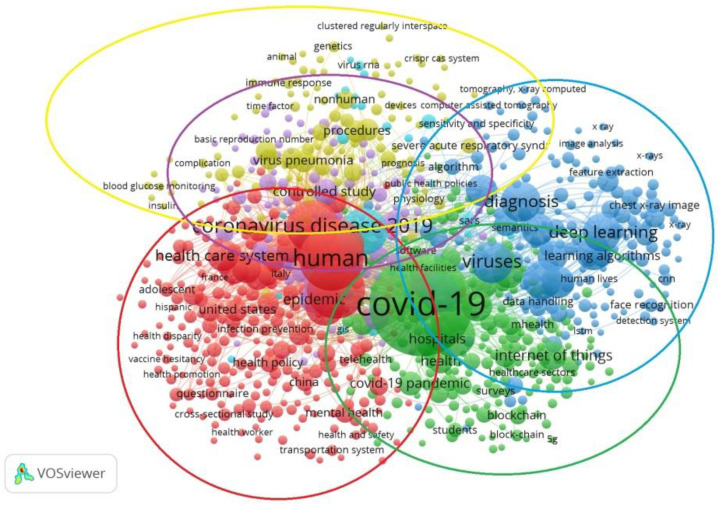
Identification of relationships between the keywords “COVID-19” and “public health system” with other concepts in scientific articles indexed by the Scopus scientometric database. Source: developed by the authors, using Vosviewer.

**Figure 2:**
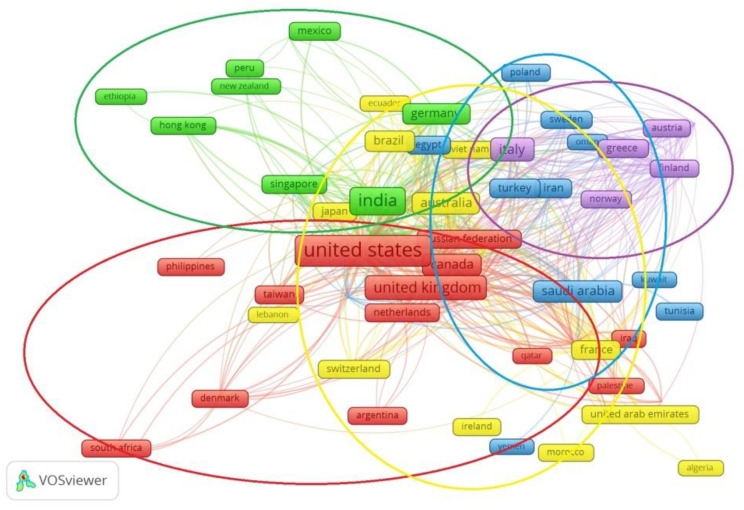
Co-authorship of scientists in scientific articles indexed by the Scopus scientometric database. Source: developed by the authors, using Vosviewer.

A bibliographic analysis of studies connecting the issues of the COVID-19 pandemic, the economic efficiency of the medical industry, and the transformation of macroeconomic development at the national level highlights the incredible popularity of these problems within the world scientific community. Therefore, it is advisable to apply generalized methods of analysis, such as research using the VOSviewer tool (Figure 1 and Figure 2), which helps to visualize bibliometric connections between publications of different databases—in particular, scientific publications indexed by the Scopus scientometric database and related to non-medical fields of knowledge, including computer science, engineering and the social sciences. A total of 1704 publications were involved in the study, and only those keywords with at least seven links among the researched ones were included in the visual presentation.

Figure 1 shows the intensity of connection between a concept and search keywords, depending on the size of the circle: the larger the circle’s diameter, the higher the frequency of mention of the corresponding concept. The most significant number of studies on the COVID-19 pandemic and health care systems focused on identifying the peculiarities of the organization of health care systems under the influence of the pandemic in different countries of the world. The next largest cluster demonstrated the great interest of world scientists in the application of blockchain technology and digitization in the medical field. Additionally, many publications focused on both medical and analytical research methods. A group of studies focused on the capabilities of health care systems to care for patients, and the readiness of medical institutions for epidemiological challenges deserved special attention. All research clusters were sufficiently interconnected and intersected, indicating the researched area’s high relevance.

The analysis of Figure 2 makes it possible to identify six groups of countries in which scientists are co-authors of publications. In particular, the first group included mainly countries United States, Canada, United Kingdom, Russian Federation, Philippines, Taiwan, Denmark and Netherlands. The second group included scientists from European countries: Austria, Cyprus, Belgium, Sweden, Serbia, Italy, Greece, Finland, etc. The third group also consisted mainly of Asian countries: China, Singapore, Hon-Kong, India, etc. The next group was transcontinental and included the Egypt, Iran, Kuwait, Oman, Poland, Romania, South Korea, Thailand etc. This shows that interest in the relationship between health care systems and the pandemic is widespread throughout the world; there is not a single continent whose representatives have not considered these questions. Transcontinental groups of co-authors prevailed, confirming this topic’s relevance.

## 3. Materials and Methods

### 3.1. Data Description

Step 1. Creation of a statistical research base

According to the basic principles of building relationships between a country’s government, its medical institutions and the population to which medical services are provided, there are several models in the world. In particular, in the Beveridge model, according to which the government of a country provides the essential social protections for the population, the foundation is a living wage and the rest is provided with the help of voluntary personal insurance [47]. In countries based on the Bismarck model, medical care is considered accessible to the majority of the population and of high quality. A feature is that half of the social insurance is paid by the employee and the other half, by the employer [48]. Additionally widespread worldwide is the model of national insurance, which is considered transitional and combines elements of the Beveridge model and the Bismarck model. Still, its advantage is the coverage of all segments of the population with medical services, the quality of which is controlled by the state. Further, there is a separate model for developing countries—characterized by a large share of personal expenses being borne by individual citizens in comparison to the share of expenses covered by the state for medical services. In this study, this model is referred to as the market model. It can be assumed that no countries are left with clean health care system models. 

Still, it is possible to conditionally single out individual countries in which one model prevails. In particular, the countries following the Beveridge model include the United Kingdom, Iceland, Ireland, Norway, Spain, Cuba, and New Zealand; Germany, Austria, Belgium, Czech Republic, France, Netherlands, and Switzerland follow Bismarck’s model; Canada, Australia, Italy, and Thailand follow the national insurance model; and China, India, Portugal, and Ukraine follow the market model [49].

To define the economic efficiency of each of the above health care systems, a set of more than 40 indicators was formed that included indicators of age distribution and birth rate (adolescent fertility rate, births per 1000 women ages 15–19, age dependency ratio, % of working-age population, crude birth rate per 1000 people); of the level of private and public expenditures (current health expenditure per capita, in current USD; domestic general government health expenditure, in % of GDP; domestic private health expenditure per capita, in current USD; external health expenditure, in % of current health expenditure; out-of-pocket expenditure, in % of current health expenditure; GNI per capita, Atlas method, in current USD); of the level of immunization of children against various diseases (immunization, DPT, in % of children ages 12–23 months; immunization, measles, in % of children ages 12–23 months; immunization, Hib3, in % of children ages 12–23 months; immunization, measles second dose, in % of children by the nationally recommended age; incidence of tuberculosis per 100,000 people; low-birthweight babies, in % of births); of the level of mortality from various causes (mortality caused by road traffic injury per 100,000 people; mortality from CVD, cancer, diabetes or CRD between exact ages 30 and 70, in %; death rate, crude, per 1000 people; mortality rate attributed to household and ambient air pollution, per 100,000 population; mortality rate attributed to unintentional poisoning, per 100,000 population; mortality rate attributed to unsafe water, unsafe sanitation and lack of hygiene, per 100,000 population; mortality rate, infants, per 1000 live births; mortality rate, under-5, per 1000 people; lifetime risk of maternal death, 1 in: rate varies by country; cause of death, by communicable diseases and maternal, prenatal and nutrition conditions, in % of total); mortality at different ages (number of infant deaths, number of deaths among children aged 5–9 years, number of deaths among youth aged 20–24 years, number of deaths of adolescents aged 15–19 years, number of neonatal deaths, number of maternal deaths, number of stillbirths, number of under-5 deaths; probability of dying among youth aged 20–24 years, per 1000 people; probability of dying among children aged 5–9 years, per 1000 people; probability of dying among adolescents aged 15–19 years, per 1000 people); provision of basic needs and attitudes of the population regarding a healthy lifestyle (people using at least basic drinking water services, urban, in % of urban population; people using at least basic sanitation services, in % of population; prevalence of current tobacco use, in % of adults; prevalence of overweight, in % of adults; fertility rate, total, births per woman); availability of medical facilities (physicians per 1000 people; nurses and midwives per 1000 people; labor force, total; hospital beds per 1000 people). The statistical base was based on data from the World Bank [50] for the years 2015–2019.

### 3.2. Multivariate Exploratory Techniques for Health System Models

The existence of a large set of indicators in research has its advantages and disadvantages. The benefits include a comprehensive overview of any problem. Still, at the same time, there is a high probability of the existence of the effect of multicollinearity, which will distort the study results. That is why there is a need to reduce and simplify the statistical base for the next steps while preserving the variance of the input array. Therefore, the principal components method was applied for the input data set; the essence of the method is to find eigenvectors and eigenvalues and rank the obtained combinations in descending order of the calculated eigenvalue. Eigenvectors are linear combinations of independent variables that influence the cumulative variance, and the corresponding eigenvalue makes it possible to estimate the weight of the influence of the corresponding vector on the total cumulative variance to find the corresponding vectors and eigenvalues from Equation (1):

(1) $$CX_{i}=\lambda X_{i}$$

C—variance-covariance matrix of the input data, $X_{i}\;$—eigenvectors, λ —scalar, eigenvalue of the corresponding eigenvector.

The number of solutions of the matrix Equation (1) equals the number of indicators of the input data, which equaled 40 for this study. The value of the total variance explained by the corresponding eigenvector is equal to the ratio of the value of the corresponding eigenvalue to the total sum of the obtained eigenvalues when solving Equation (1). Taking into account the cumbersome calculations of the forty-dimensional matrix, we applied the principal components method using the Statistica Portable software; namely, the Multivariate Exploratory Techniques/Principal Components and Classification Analysis module for each health system model, was used to ensure. With the provision of cumulative variation at the level of not less than 75%, the number of factors that it provides was selected for each model. For countries in which the Beveridge model prevails, the cumulative variation was provided by three factors at the level of 82.5%; for the Bismarck model, there were also three factors and 81.5% of the variability; for countries following the transitional model of national insurance, two factors were enough to ensure 88.6% of the variation; for the market model, two factors—77.5%.

Based on the determined required number of factors for each model separately, the contribution of each indicator was calculated. The results of the eigenvalues of the correlation matrix are presented in Table 1, which is used to characterize the priority of the indicators.

Table 1 shows the values of the specific weight for each factor and the number selected for each model using the cumulative variance estimation. The total value of weights in each row of the table equals 100%, and the higher the indicator’s value, the more significant contribution it makes to the overall variance in its model. Due to the defined weights of each indicator of the relevant factor in a particular model, it is possible to select the appropriate indicator; we will use Criterion (2):

(2) $$\frac{\sum_{i=1}^{k}x_{i}\cdot f_{\mathrm{ji}}}{\sum_{i=1}^{k}x_{i}}\geq 0.7$$

where k—number of factors, $x_{i}$—variation of the i-factor indicator; $f_{\mathrm{ji}}$ is the specific weight of the i-factor.

In the applied Criterion (2) results, all four models were matched with indicators showing the level of mortality due to the lack of basic sanitary standards and water, as well as the number of child deaths and air quality. For the three models, in addition to the market one, the indicators that maximally describe the dispersion variability are the birth rate and labor force volumes. In particular, the sets of indicators for the Bismarck and Beveridge model turned out to be similar: indicators of external and internal costs for medicine, the level of staffing of medical institutions and the percentage of the population addicted to harmful habits. For the market model, indicators of child injuries, the lack of essential sanitary hygiene products, the percentage of women dying during childbirth and the number of deaths among children turned out to be fundamental. The transitional model includes mortality rates among children (adolescents and newborns from infectious diseases), rates of tuberculosis, injury rates among the population, the percentage of women dying during childbirth and the number of deaths among children.

### 3.3. Construction of Integrated Efficiency Indices and Indicators of Resistance to COVID-19 of the Model of the Public Health Care System

The obtained indicators for each model have an extensive range of data, which can negatively affect the next steps. Therefore, normalization methods were used to bring all data to a comparable form. At the same time, Formula (3)—minimax normalization—was applied for indicators that have a positive effect on the overall efficiency of the model, and Procedure (4) was used for system destructors, the majority of which are:

(3) $$x_{i}{}^{*}=\frac{x_{i}-\underset{j}{\min}x_{j}}{\underset{j}{\max}x_{j}-\underset{j}{\min}x_{j}}$$

(4) $$x_{i}{}^{*}=1-\frac{x_{i}-\underset{j}{\min}x_{j}}{\underset{j}{\max}x_{j}-\underset{j}{\min}x_{j}}$$

where $x_{i}{}^{*}$—normalized i-th value of the indicator, $x_{i}$—current value, $\underset{j}{\min}x_{j}$ Ta $\underset{j}{\max}x_{j}$—the minimum and maximum values of the j-indicator, respectively.

So, as a result of applying Formulas (3) and (4), the indicators were reduced to a comparative form in the range from 0 to 1, where 1 corresponds to the best value of the variable and 0 to the worst.

Let us consider the economic efficiency of the country’s health care models as an integral indicator of the ratio of the resources spent in the medical sphere to the useful results obtained and compare it with the efficiency results according to the DEA analysis. The integral indicator includes the determinants indicating the effectiveness of the medical system itself (birth and death rates, child vaccination rates, etc.) and economic indicators: public and private expenditures on health care, meeting the basic needs of the population, the ability of the country’s economy to maintain the required number of medical experts (doctors, nurses), hospital beds.

Values for each country for each indicator of the relevant factors of the corresponding model must be combined into an integrated health system efficiency index (EHS) using additive Convolution (5). Together with the integral index of the effectiveness of the model of the public health care system, the index of resistance to the pandemic (PR) was calculated according to Formula (6) and the generalized average for each model:

(5) $${EMS}_{j}=\frac{\sum_{i=1}^{n}x_{i}^{*}{}_{j}+\underset{j}{\min}\sum_{i=1}^{n}x_{\mathrm{ij}}^{*}+\sigma \left(\sum_{i=1}^{n}x_{\mathrm{ij}}^{*}\right)}{\underset{j}{\max}\left(\sum_{i=1}^{n}x_{\mathrm{ij}}^{*}+\underset{j}{\min}\sum_{i=1}^{n}x_{\mathrm{ij}}^{*}+\sigma \left(\sum_{i=1}^{n}x_{\mathrm{ij}}^{*}\right)\right)}\cdot 100\%$$

(6) $$\mathrm{PR}=\frac{{\mathrm{Inf}}_{j}-{\mathrm{Death}}_{j}}{{\mathrm{Inf}}_{j}}\cdot 100\%$$

where $\underset{j}{\min}\overset{n}{\underset{i=1}{\sum }}x_{\mathrm{ij}}^{*}$—the minimum value of the time series for j—country, i—indicator, σ—standard deviation of the corresponding indicator, ${\mathrm{Inf}}_{j}$—the number of officially registered persons infected with COVID-19 in country j on 2 October 2022 [51], Death—the number of officially registered persons who died as a result of the COVID virus.

### 3.4. DEA Analysis

With the help of software (Banxia Frontier Analyst 4), an evaluation of the effectiveness of health care system models was carried out, according to the principles of fractional-rational programming of the initial approximate CCR model, the goal of which is to maximize the values of the initial parameters. The most effective model will be considered to have the closest approximation of the initial parameters (when constructing the isoquant) to the ideal state and corresponds to Formula (7) [52]:

(7) $$\mathrm{maxEMS}=\frac{\sum_{k}u_{k}y_{k}}{\sum_{l}v_{l}x_{l}}\;;\;\{\begin{array}{c} \frac{\sum_{k}u_{k}y_{k}}{\sum_{l}v_{l}x_{l}}\leq 1\\ u_{k},v_{l}\geq \varepsilon \end{array}\{\begin{array}{c} \frac{\sum_{k}u_{k}y_{k}}{\sum_{l}v_{l}x_{l}}\leq 1\\ u_{k},v_{l}\geq \varepsilon \end{array}$$

where EMS—the level of effectiveness of the health care system; $u_{k}$ and $v_{l}$—specific weight of the k-th indicator of the category of conditional outputs and the l-th indicator of conditional inputs, respectively.

## 4. Results

**Figure 3:**
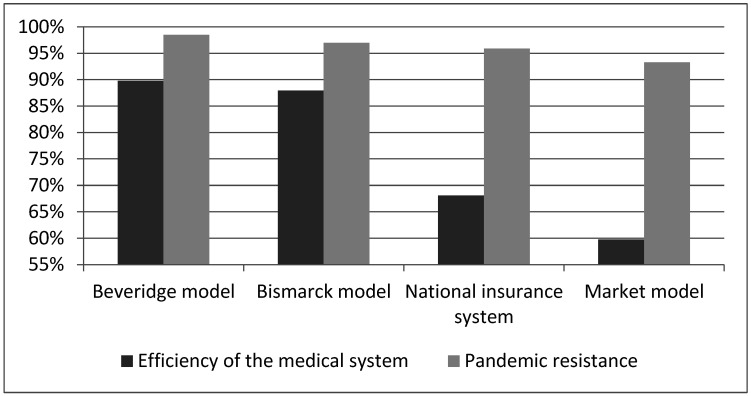
Comparison of the results of the effectiveness of the medical system and resistance to the pandemic according to the model of the organization of the health care system. Source: constructed by the authors.

Therefore, for a comprehensive comparison of models of the health care system, integral indices indicated the economic efficiency of the model (Figure 3), resistance to the effects of the COVID-19 pandemic (Figure 3) and the calculated efficiency of the model based on frontier DEA analysis (Table 2).

Therefore, the analysis of the results calculated for each model of the health care system, both the value of the efficiency of the medical system and resistance to the COVID-19 pandemic, allowed us to conclude that the system built according to the Beveridge principle turned out to be the best, because it had the highest efficiency rate of 90% and a pandemic resistance rate of 98%. Next in quality was the system built according to the Bismarck principle, because it had an 88% efficiency index in the medical field and 97% resistance to the pandemic. So, with a decrease in economic efficiency by 2%, it follows that 1% of the country’s population was less protected from the harmful effects of the spread of the disease. In third place was the model built on the principle of national insurance, with the corresponding results of 68% and 96%. In last place was the market model (60/93). This confirms the hypothesis that there is a direct relationship between the effectiveness of the health care system and the ability to respond quickly to epidemiological challenges.

So, according to the frontier analysis, the Beveridge model was also the most effective, with an average efficiency value of 96.6%. Moreover, four of the seven countries studied in this group had the maximum level of efficiency—100% (Ireland, Iceland, New Zealand and Great Britain). Norway had the worst indicators in the group—84%, but this value exceeded the average values for all countries. According to the frontier analysis, the second place in terms of efficiency of the economic component was held by countries in which a health care system model based on the principle of national insurance had been implemented, at the level of 95.5%. All countries following this model had high levels of efficiency, greater than 92%, and Canada had an ideal state. The third place belonged to the Bismarck model, according to the results of the DEA analysis—73.7%, and the Czech Republic had perfect results. According to both the integral indicator and the results of the construction of the CCR model, the market model of the organization of the health care system, according to which most of the funds spent on medical services are from personal savings of the population, had the worst performance results.

Further, we will take a closer look at the results of the frontier analysis and focus on the largest deviations from the ideal state for each indicator. A positive percentage value meant that a country had a reserve of using the indicator to a specified extent (in %). This meant that country could preserve the same efficiency level even if the indicator changed. A negative percentage meant that the country under study should reduce this indicator by a specified extent (in %) to bring the efficiency value closer to 100% in terms of the relevant health care organization model.

In particular, according to the results of the construction of the CCR model, Great Britain, New Zealand, Iceland and Ireland had an ideal state of economic efficiency of their medical care systems in the Beveridge model. This meant that all of the studied indicators were in relatively perfect condition. However, the remaining countries had to adjust their actions to achieve maximum efficiency. For example, Cuba and Spain did not reach the threshold due to poor maternal mortality (−7%/−11%) and deaths related to unsafe water, unsafe sanitation and lack of hygiene (−10%/−3%). Reforming the Norwegian medical system should aim to increase domestic private spending on health care (+10%), as well as increase the supply of medical facilities with doctors (+15%) and hospital beds (+21%).

The analysis of the obtained data according to the Bismarck model indicated that only the Czech Republic achieved the ideal state and did not need to reform its medical system. For example, the government of Belgium needed to reduce the risk of maternal mortality (−23%) and the probability of child mortality (−17%); in addition, it was necessary to increase the supply of medical facilities with doctors (+9%) and hospital beds (+11%). As for Austria, it was recommended that it revise its policies to reduce the mortality rate among the population (−24%), and in particular, to prevent infant mortality of children under 5 years of age (−36%). In addition, there was a need to increase expenditures on medicine (+11%). In France, it was found to be necessary to reduce the dependency ratio of the population (−27%) and to reduce the mortality rate among children under 5 years of age (−22%), but to ensure an increase in the supply of medical institutions with doctors (+13%) and hospital beds (+10%). The Netherlands also had shortcomings in the mortality rate of children under 5 years of age (−22%) and their mothers (−12%); in addition, the mortality rate due to unsafe water was quite high (−14%) and required an increase in the provision of medical facilities with doctors (+8%)) and hospital beds (+28%). Germany had fewer problematic indicators, but it was necessary to take care of the increase in costs (+15%) and the increase in medical personnel (+16%).

Among the countries based on the transitional model of national insurance, only Canada had reached the ideal state; the rest of the studied countries needed to reform their medical service systems. In particular, Australia, although approaching the perfect state, needed to take care of the increase in medical expenses (+5%) and the increase in the percentage of the labor force (+18%). Italy needed a significant reduction in the mortality rate of children aged 5–9 years (−22%) and an increase in the birth rate (+20%). In Thailand, it was necessary to reduce the mortality rate due to poor-quality water (−31%), due to tuberculosis (−21%) and maternal mortality (−19%).

According to the results of the frontier analysis of the health care system market model, only China’s medical system had reached the limit of ideal values, and the rest of the countries needed to carry out reforms. In particular, in India and Ukraine, significant reductions in the child (−35%/−20%) and maternal (−23%/−24%) mortality were needed, in addition to a reduction in morbidity due to poor air quality (−10%/−35%) and tuberculosis (−12%/−23%). Separately, Ukraine should take care of increasing the birth rate (+56%). Regarding Portugal, it was also necessary to pay attention to maternal mortality (+15%) and to increase the percentage of people who have the opportunity to use essential sanitary services (+8%).

## 5. Discussion

Ukraine, India and Portugal proved to be the least prepared for the challenges of the pandemic and demonstrated the lowest values of integrated indices of economic efficiency. These countries are either on the path to transformation or based on a market model with direct payments for medical services, i.e., most of the medical services are paid for by the population. As a result, these countries turned out to be the worst prepared for a quick response to epidemiological threats with the help of early detection of the first signs of disease, availability of medical services for the majority of the population, sufficient equipment for hospitals to provide quality service, high qualification of doctors, etc.

Regarding the resilience to pandemic challenges according to the health care model, the Beveridge model showed the best performance (98.9%), followed by the Bismarck model (97.9%) and the national insurance model (97.8%); the market model showed the worst results (97.2%). On the one hand, the averages indicated sufficient proximity between the models and showed slight variation, so we can conclude that the choice of model is not crucial for epidemiological threats. However, if we calculate the percentages, we can see hundreds of human deaths behind each tenth of a percent that might have been avoided if the right health care model had been chosen within the country. That is why we carried out this comparative study, which may be useful for those countries undergoing health care reform.

Therefore, the Beveridge model proved to be the most effective according to various methodologies, according to which the share of medical institutions is public, together with their payment for medical services, which is paid for by taxes. Since medical institutions are under state control, there is equal access to medical services for different population strata. This model was also characterized by the economical use of funds and state control over the quality of medical services. The main disadvantage of this model is the health care system’s inertia with regard to innovations and efficiency improvements. In other words, it turns out that to meet the challenges of the pandemic, maximum coverage of all population strata with basic health services became more urgent than service quality. This may not work out under other circumstances, of course, if the infection spreads at a slower rate and has other consequences. Beveridge’s model is not a panacea for all future epidemiological challenges; this study only tested its effectiveness during the COVID-19 pandemic and provided recommendations for countries currently undergoing medical system reform.

## 6. Conclusions

The conducted comparison of the efficiency of models of the organization of the world health care system with the use of frontier DEA analysis and calculated integral indices showed that the most effective model of the construction of the health care system was the Beveridge model. Most countries that had implemented it had the most significant values of integral indices, calculated using the additive convolution of normalized relevant indicators of the medical field. The results for the economic efficiency of health care systems, according to the constructed CCR model, confirmed the proposed hypothesis at the level of 97%. The average value of the integral index of economic efficiency for the Beveridge model ensured the quality of the model because its value was also the largest compared to other models and equaled 90%. The Bismarck model and the national insurance model were next in the ranking of effective models according to these indicators, and the results for them differed: according to the calculated integral indices, the Bismarck model was better, and according to the results of the frontier analysis, the national insurance model was better.

Therefore, the recommendation for reforming the health care system of Ukraine, Portugal, and India is to review the transformation and reform scenarios based on model countries (Great Britain, Iceland, Ireland, New Zealand, China, the Czech Republic, Canada), and the most effective model—the Beveridge model. In particular, the organization of the health care system of Ukraine is already in a state of transition from a market model to a combination of the principles of the Beveridge model and the national insurance system. However, the previous system still had a negative impact that influenced the medical field’s unpreparedness to respond promptly to the challenges of the pandemic in 2020. So, this study confirms that Ukraine is moving in the right direction of reforming its medical system; it is only necessary to speed up the pace.

## Figures and Tables

**Table 1:** Case contributions, based on correlations in the section of each factor (fragment).

	Beveridge Model			Bismarck Model			Transitional Model		Market Model	
	Factor 1	Factor 2	Factor 3	Factor 1	Factor 2	Factor 3	Factor 1	Factor 2	Factor 1	Factor 2
Adolescent fertility rate (births per 1000 women ages 15–19)	0.0610	0.0840	0.0352	0.0540	0.1002	0.0235	0.0532	0.0615	0.0533	0.0438
Age dependency ratio (% of working-age population)	0.0608	0.0803	0.0399	0.0540	0.0969	0.0181	0.0532	0.0577	0.0532	0.0368
Birth rate. crude (per 1000 people)	0.0610	0.0834	0.0335	0.0540	0.0997	0.0244	0.0532	0.0605	0.0533	0.0436
Current health expenditure per capita (current USD)	0.0384	0.3579	0.1778	0.0505	0.6869	0.7191	0.0523	0.6213	0.0528	0.4044
…	…	…	…	…	…	…	…	…	…	…
Physicians (per 1000 people)	0.0610	0.0841	0.0320	0.0540	0.1000	0.0253	0.0532	0.0610	0.0533	0.0440
Probability of dying among adolescents ages 15–19 years (per 1000)	0.0610	0.0843	0.0314	0.0540	0.1003	0.0257	0.0532	0.0614	0.0533	0.0447

**Table 2:** Results of the frontier analysis for different countries of the world according to the model of the health care system (%).

Country	Beveridge Model	Country	Bismarck Model	Country	Transitional Model	Country	Market Model
Cuba	97	Belgium	72	Canada	100	China	100
Iceland	100	Austria	65	Australia	97	India	44
United Kingdom	100	Czech Republic	100	Italy	93	Portugal	85
Norway	84	Switzerland	62	Thailand	92	Ukraine	45
Spain	95	France	66				
Ireland	100	Germany	92				
New Zealand	100	Netherlands	59				
Average	96.6	Average	73.7	Average	95.5	Average	68.5

## Data Availability

Not applicable.
